# Prioritizing WHO normative work on maternal and perinatal health: a multicountry survey

**DOI:** 10.1186/1742-4755-8-30

**Published:** 2011-10-28

**Authors:** Cordelia EM Coltart, João Paulo Souza, Ahmet M Gülmezoglu

**Affiliations:** 1 UNDP/UNFPA/WHO/World Bank Special Programme of Research, Development and Research Training in Human Reproduction, Department of Reproductive Health and Research, World Health Organization, Geneva, Switzerland

**Keywords:** Prioritization, multi-country survey, maternal health guidelines, perinatal health guidelines, guideline development

## Abstract

**Background:**

WHO develops evidence-based guidelines for setting global standards and providing technical support to its Member States and the international community, as a whole. There is a clear need to ensure that WHO guidance is relevant, rigorous and up-to date. A key activity is to ascertain the guidance needs of the countries. This study provides an international comparison of priority guidance needs for maternal and perinatal health. It incorporates data from those who inform policy and implementation strategies at a national level, in addition to targeting those who use and most need the guidance at grassroot level.

**Methods:**

An online multi-country survey was used to identify WHO guidance priorities for the next five years in the field of maternal and perinatal health. WHO regional and country offices were requested to respond the survey and obtain responses from Ministries of Health around the world. In addition, the survey was disseminated through other networks and relevant electronic forums.

**Results:**

A total of 393 responses were received, including 56 from Ministries of Health and 54 from WHO/UN country offices. 75% of responses were from developing countries and 25% from developed countries. Guidance on strategies focusing on 'quality of care' issues to reduce all-cause maternal/perinatal mortality was considered the most important domain to target, which includes for instance guidance to improve access, dissemination, implementation of effective practices and health professionals' education.

**Conclusions:**

This study provides a panorama of international priority guidance needs for maternal and perinatal health. Although clinical guidance remains a priority, there are other areas related to health systems guidance, which seem to be even more important. Overall, the domain ranked highest in terms of greatest need for guidance was around quality of care, which included questions related to educational needs, access to and implementation of guidance.

## Introduction

Improving maternal and newborn health is a key area of work for the international health community and especially for the World Health Organization (WHO). Since the Millennium declaration in 2000 and the establishment of the Millennium Development Goals, the focus on improving maternal and newborn health has intensified. In this context, the recently launched United Nations *Global strategy for women's and children's health (*September 2010) is providing an added impetus to the effort of saving more than 16 million women and children over the next four years [1].

A core WHO activity is the development of evidence-based guidelines for setting global standards and providing technical support to its Member States. WHO member states and the international community place great importance on WHO guidelines to inform their policies and practices. Therefore, it is essential that WHO guidance is relevant, high-quality and up-to-date. Since 2007, WHO has established a Guidelines Review Committee to oversee the process of developing evidence-based recommendations and subsequently the WHO guideline development process has become more rigorous [2]. However, WHO departments are often challenged regarding the prioritization of topics for guideline development and whether the selected topics accurately represent the 'demand' from the field.

Since the mid-nineties, WHO normative work on maternal and perinatal health has focused on clinical guidance, particularly aimed at peripheral, small hospitals or primary care facilities [3]. Conversely, WHO normative work on newborn and infant health has focused on both primary care and community-level guidance [4]. While clinical research has provided some effective interventions to reduce maternal morbidity and mortality, there are notable challenges in getting those interventions implemented across all levels of the health system, including finding innovative solutions to providing access to effective care in the community. Furthermore, it has become increasingly apparent that the organizational or system-wide challenges in implementing effective practices are hugely important [5-8].

Against this background, WHO launched a project in 2009 to address knowledge synthesis, exchange and translational issues in sexual and reproductive health in a systematic manner. This project has the acronym GREAT which stands for **G**uideline development, **R**esearch prioritization, **E**vidence synthesis, **A**pplicability of evidence and **T**ransfer of knowledge[9]. As part of the GREAT project, a key initial activity was to ascertain the specific guidance required by countries. In connection with that, this report presents the findings of an international survey conducted by WHO in 2010. In addition to the clinical guidance related questions, information was sought regarding the broader issues, such as the type of provider, place of care, quality of care and the need for educational interventions. Our primary focus was to obtain responses from WHO counterparts in the countries, such the relevant authorities in the Ministries of Health (MOH) of Member States and the UN agencies country level staff. We also targeted other stakeholders, such as clinicians, programme managers and other bilateral or international organization staff active in the field. The overarching question we asked was "*what are the priority topic areas of guidance required from WHO to support the reduction of maternal and perinatal mortality and morbidity"*. In addition to the online survey, virtual global discussion forums and targeted focus group discussions were carried out. This paper summarizes the process and focuses primarily on the output of the multicountry survey.

## Objective

To identify WHO guidance priorities for the next five years in the field of maternal and perinatal health (in 2010).

## Methods

This analysis is based on a survey administered electronically between 6 August - 17 September 2010. The questionnaire was developed in collaboration with the input of WHO regional offices. Preceding the survey administration, an online community named "WHO Guidance" was created in the WHO Implementing Best Practices Knowledge Gateway [10]. The launch of the community was advertised using pre-existing e-mail lists and other relevant established online communities. A two-week online discussion was convened among the "WHO Guidance" community. This discussion focussed on the same issues that would be covered in the survey, but in a narrative way. At the conclusion of the discussion, an electronic link to the survey was provided with a request for participation to all members of the community. Following this, a memorandum was sent to every WHO country office (except the 21 countries in the EMRO region where the regional office disseminated the survey), with the same instructions. The memorandum included the request for replies to be sought from the Ministry of Health of each country directly, with further dissemination of the survey as widely as possible to hospitals, clinics, research institutions and other health care professionals. In addition, the survey was circulated through several maternal and child health online forums run both within the WHO and externally through such organisations as the HIFA (Health Information for All) 2015 and HIFA-CHILD groups.

The survey was designed to cover all domains, in which WHO undertake work related to improving maternal and perinatal health, grouped in to five key areas: health systems (where care is provided), finance and procurement, human resources (who provides the care), quality of care and clinical guidelines. The questionnaire included a total of 55 questions, across these five domains. Each category included between five and nine specific (lower level) questions and the survey requested participants to rank the importance of WHO guidance to these issues on a scale of 1 to 9, with 1 being "not important" and 9 equating to "very important". The final question involved ranking the five domains in order of importance (1 = highest to 5 = lowest).

The survey was only available in the English language. It was initially piloted among RHR staff members, who internally discussed and revised, providing feedback to the main investigators. Once the questionnaire was applied, all data were collated and analyzed by one researcher (CEMC), and reviewed by two other researchers (JPS/AMG). Any category scoring above 7 was considered important for further evaluation within WHO. All data were included except if multiple entries were entered by the same individual. Entries were excluded if no quantitative data were provided. SurveyMonkey was the online electronic survey tool used in this study (SurveyMonkey - http://www.surveymonkey.com).

## Results

**Figure 1:**
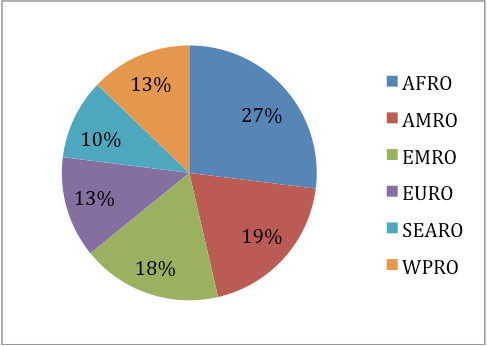
**Responses by WHO region (AFRO/AMRO/EMRO/EURO/SEARO/WPRO)**.

There were a total of 393 responses, with 300 meeting the inclusion criteria for analysis. 95.5% of respondents stated their organisational affiliation. Responses were received from 56 Ministries of Health and 54 WHO/UN country offices. 56% of participants were involved in health at a national, 27% at regional and 30% at a global level. 97.9% declared their country: 75% of all responses were from 'developing' and 25% from 'developed' countries as defined by International Monetary Fund and the World Bank [11]. Over a quarter of all responses analyzed were from the AFRO Region, with AMRO and EMRO accounting for nearly 20% of responses each (Figure 1). Responses were received from ten francophone countries and five Spanish speaking countries.

A total of 15 questions ranked greater than 8 overall (Table 1) and 43 questions were considered 'important' ranking over 7. Guidance on strategies focusing on 'quality of care' issues to reduce all-cause maternal/perinatal mortality was considered the most important domain to target across all subgroups. This included guidance to improve dissemination, implementation, education and information sharing. Strategies focusing on the care provider and who/how to provide care ranked second overall, across all domains (e.g. guidance around defining and expanding the roles of each cadre of health care professional and strategies to mobilize community care). Strategies focusing on where the care is provided and clinical guidance ranked closely at third and fourth, respectively. Strategies focusing on finance and procurement ranked lowest (5^th^) across all subgroups (Table 2).

**Table 1:** Top 15 guidance priority questions (Overall, WHO and MOH rankings).

Overall ranking n = 300	Overall SCORE	QUESTION	WHO ranking n = 54	MOH Ranking n = 56
1	8.37	Early neonatal interventions for treatment of asphyxia (e.g. resuscitation)	1	3
2	8.33	PPH treatment in facilities	4	5
3	8.25	Strategies to increase contraceptive use after abortion	14	21
4	8.24	Pre-eclampsia treatment interventions (e.g. magnesium sulfate, antihypertensives, others)	2	10
5	8.22	Eclampsia treatment interventions	3	13
6	8.22	PPH prevention in facilities	11	15
7	8.19	Guideline dissemination and implementation strategies	12	2
8	8.19	Timing of delivery (labour initiation, early-onset severe disease)	7	16
9	8.18	Strategies to ensure access to prompt treatment for complications of spontaneous and unsafe abortion	6	20
10	8.07	Effects of using clinical guidelines/care pathways	10	1
11	8.07	Strategies to increase the large scale implementation (scaling up) of effective practices at the state or country level	5	18
12	8.07	Interventions for increasing the proportion of health professionals practicing in rural and other underserved areas	8	27
13	8.04	Interventions to improve referrals from primary to secondary level health care	9	9
14	8.03	Strategies for integrating primary health services at the point of delivery in low and middle income countries (LMIC)	25	19
15	8.02	Prevention of preterm birth	22	11

**Table 2:** Overall ranking based on guidance domain (1 = highest, 5 = lowest)

Category	Overall Ranking n = 300	WHO n = 54	MOH n = 56
Reducing all-cause maternal/perinatal mortality: Strategies focusing on the QUALITY OF CARE	1	1	1
Reducing all-cause maternal/perinatal mortality: Strategies focusing on the CARE PROVIDER	2	2	2
Reducing all-cause maternal/perinatal mortality: Strategies focusing on WHERE the care is provided	3	4	4
CLINICAL guidance to reduce maternal and perinatal morbidity and mortality	4	3	3
Reducing all-cause maternal/perinatal mortality: Strategies focusing on FINANCE/PROCUREMENT issues	5	5	5

## Discussion

This study provides an international comparison of priority guidance needs for maternal and perinatal health. Although clinical guidance remains a priority, there are other areas related to health systems guidance, which seem to be even more important. Overall, the domain ranked highest in terms of greatest need for guidance was around quality of care, which included questions related to educational needs, access to and implementation of guidance.

The translation from evidence to real practice is complex and effective implementation of guidance at community grassroot levels remains difficult. Barriers to implementation include language, lack of education, lack of human resources, resource constraints and lack of incentives. A key priority should be put in place to ensure optimal, effective dissemination and transfer of existing, evidence-based knowledge into health systems through the use of guidelines. Although WHO guidelines are freely available on the internet and free hard copies can be requested, the existence and availability are often not well known, particularly to those who need them the most, for example in the many settings where access and ability to use the internet is limited. Dissemination to grassroots levels should be a priority, as is translation into local native languages to maximize uptake. Given the varying contexts between countries, it is clear that implementation research that incorporates both quantitative and qualitative aspects of increasing the utilization of effective practices is a priority.

The international respondents in this survey expressed a need for evidence-based guidance to define the roles of health care professionals, particularly in relation to task-shifting e.g. expansion of roles of nurses, midwives and traditional birth attendants (TBA), in order to provide access to a greater population. The need for guidance regarding TBA training was highlighted both in the survey and the online discussion preceding the survey. This discussion was particularly polarized with some participants being opposed to any involvement of TBAs, while others suggested that in many places TBAs are the only group available to deliver maternal and infant care (World Health Organization, Online Discussion on the roles of TBAs in achieving MDGs 4 and 5, Day 5, available at http://my.ibpinitiative.org/whoguidance/Optimize4MNH). In this context, it may be sensible to offer training to TBAS as opposed to leave them completely outside any regulated system. Financial issues rated as the lowest priority across all respondents, which may reflect the professional orientation of the survey respondents.

Interestingly, the responses from WHO/UN offices and MOH did not always reflect those from other stakeholders, primarily clinicians and researchers. For example, strategies to increase contraception after birth ranked highly among other stakeholders, but was not considered a priority by opinion formers or policy makers in national positions at WHO and MOH (Table 1). Of note, the 1st and 2^nd ^priority ranking among WHO country offices are directed at specific clinical interventions, while the MOH priorities reflect more emphasis on local implementation of guidelines. WHO and MOH both considered guidance on implementation of mechanisms to expedite referral from primary to secondary care as an important need (rank 9th).

The main strengths of this survey lie in the data obtained from MOH and WHO country offices. Data were obtained directly from those who inform policy and implement strategies at a national level. They are well placed to assess the guidance needs, with an experiential view of current practices. Additionally, this is reinforced by the inclusion of data obtained from those countries where maternal and perinatal mortality is high.

Whilst much rich data can be extracted from this survey, there are limitations associated with the intrinsic difficulties in undertaking such a study. It was not practical to establish a sampling frame for the 'other stakeholder' respondents, although for the MOH and WHO/UN country staff, we aimed to get responses from all member states. The survey was circulated in the English language, therefore significantly limiting the responders and possibly introducing some selection bias. However, a substantial proportion of the responses came from non-English speaking countries. Moreover, the online nature of the survey hindered those with little or no access to the internet. Of note, developed countries with ease of access to the internet only contributed 25% of the results. However, in developing countries, guidance is a higher priority, as access to other information is scarce. Therefore, WHO guidance on maternal and child health is considered 'more relevant' to the developing world setting, with a need for robust local guidance. An explanation to account for the positive response rate from developing countries may be the motivation of health professionals in areas with limited access to evidence, to support initiatives to provide better guidance.

Furthermore, due to the snowballing technique used, it is impossible to track the overall dissemination of the survey. All WHO country offices were sent identical information, but it is unclear which offices followed the requests and brought the survey to the attention of the MOH. Interestingly, more MOH replied than WHO country offices, although the geographical nature of responses was closely mirrored between these two subgroups.

## Conclusions

There is a clear need to ensure that WHO guidance is relevant, rigorous and up-to-date. The online discussion and the survey outlined here give useful pointers about future orientation of WHO guidance and normative work in general. It is important that these types of studies are repeated with improved methodology and perhaps more regional focused qualitative or quantitative studies.

## List of abbreviations

AFRO: African regional office; AMRO: Americas regional office; EMRO: Eastern Mediterranean regional office; EURO: European regional office; GREAT: Guideline development, Research prioritization, Evidence synthesis, Applicability of evidence and Transfer of knowledge (WHO project); HIFA: Health Information for all; MOH: Ministry of Health; PPH: Postpartum haemorrhage; SEARO: South East Asian regional office; TBA: Traditional birth attendants; UN: United Nations; WHO: World Health Organization; WPRO: Western Pacific regional office.

## Competing interests

The authors declare no conflict of interests. The views expressed in this paper are those of the authors as individuals, and they not necessarily represent the views of the World Health Organization.

## Authors' contributions

All three authors CEMC, JPS, AMG, involved in the conception, design, acquisition of data, analysis/interpretation. CEMC drafted this manuscript, all authors contributed and approved the final version.
